# Low Prevalence of HIV-1 Antiretroviral Resistance Among Patients Experiencing Virological Failure on Dolutegravir-Based Regimens: A Prospective Cohort Study in Bunia, Democratic Republic of the Congo

**DOI:** 10.3390/tropicalmed11100286

**Published:** 2026-10-10

**Authors:** Alex Liripa Kwendra, Augustin Mouinga-Ondeme, Jéordy Dimitri Engone-Ondo, Herman Chelo Ngadjole, Otniel Adjala-Ondeme, Oloumbou Fedricksen Eldridge, Salomon Batina Agasa, Ivan S. Mfouo-Tynga

**Affiliations:** 1 Département de Médecine Interne, Infectiologie Tropicale, Faculté de Médecine, Université de Bunia, Bunia 292, Democratic Republic of the Congo; dralexliripa1@gmail.com; 2 Programme National de Lutte Contre le VIH/SIDA-IST-Hépatites Virales, Bunia 185, Democratic Republic of the Congo; 3 Unité des Infections Rétrovirales et Pathologies Associées, Centre International de Recherches Médicales de Franceville (CIRMF), Franceville P.O. Box 769, Gabon; ondeme@yahoo.fr (A.M.-O.); engonejordy@gmail.com (J.D.E.-O.); otadjala@gmail.com (O.A.-O.); eldridgefedricksenoloumbou@gmail.com (O.F.E.); 4 Département de la Santé Publique, Université de Goma, Goma 2204, Democratic Republic of the Congo; chhermangbea@yahoo.fr; 5 Département de Médecine Interne, Universités de Kisangani, Kisangani 2012, Democratic Republic of the Congo; agasasalomon@gmail.com

**Keywords:** HIV-1 drug resistance mutations, dolutegravir, virological failure, genotypic sensitivity score, Bunia-DRC

## Abstract

The adoption and widespread use of dolutegravir (DTG)-based antiretroviral therapy (ART) are key strategies for achieving global 2030 targets, though emerging resistance threatens long-term success. This study evaluated HIV-1 drug resistance mutations among participants experiencing virological failure in Bunia, Democratic Republic of the Congo (DRC). Over an 11-month period, a prospective cohort study was conducted across 20 healthcare facilities in Bunia, Ituri Province. Targeted genotyping of the HIV-1 pol gene was performed, antiretroviral resistance mutations were identified, and genotypic sensitivity scores (GSSs) were calculated using the Stanford HIV Drug Resistance Database. Virological failure occurred in 12.3% (74/603) of participants, and targeted genotyping of the HIV-1 pol gene was attempted on 53 samples. This subgroup comprised 41 females (55.4%) and 33 males (44.6%), with a median age of 36 years (IQR: 17.0) and a median ART duration of 24 months. Subsubtype A1 predominated (38%), followed by subtypes G (26%) and D (9%); recombinant forms, led by CRF02_AG, accounted for 16%. Mutations associated with resistance to reverse transcriptase inhibitors were detected in 26.4% (14/53) of successfully sequenced samples, compared with 33.9% (18/53) for integrase strand-transfer inhibitors. Overall, GSS analysis revealed that 98% (50/51) of evaluable participants scored 3, whereas only 2% (1/51) scored below 3. This study demonstrates that DTG-based ART remains highly effective in Bunia. To optimize treatment outcomes and support progress toward the UNAIDS 95-95-95 targets, targeted interventions to enhance treatment adherence are urgently warranted.

## 1. Introduction

The human immunodeficiency virus (HIV) remains a critical global public health challenge [1]. According to UNAIDS estimates published in 2026, approximately 92 million people have acquired HIV since the start of the epidemic, with 42.2 million AIDS-related deaths to date and active transmission persists across all regions worldwide [1,2].

In 2025, approximately 41 million people were living with HIV globally, with 32.1 million accessing antiretroviral therapy (ART) [1,2]. As part of the global strategy to end AIDS as a public health threat by 2030, United Nations Member States adopted the 95-95-95 targets for HIV diagnosis, treatment coverage, and viral suppression [1,2]. The scale-up of ART to 89% coverage by 2025 has driven dramatic reductions in HIV-related morbidity and mortality [2]. However, the long-term sustainability of these gains is threatened by the emergence of drug-resistant viral variants [3,4]. Surveillances conducted between 2014 and 2021 across 38 countries worldwide documented significant resistance to nucleoside/nucleotide reverse transcriptase inhibitors (NRTIs/NtRTIs), non-nucleoside reverse transcriptase inhibitors (NNRTIs), and protease inhibitors (PIs) [5].

Simultaneously, the extensive genetic diversity of HIV remains one of its defining characteristics, influencing global distribution, treatment outcomes, disease progression, and the selection of drug-resistant strains [6]. Indeed, the emergence of drug resistance mutations (DRMs) poses a direct threat to achieving the third UNAIDS target 95% viral suppression among individuals on treatment by driving virological failure in people living with HIV (PLHIV) receiving antiretroviral therapy (ART) [7,8]. In response to rising baseline resistance, the World Health Organization (WHO) has recommended dolutegravir (DTG), a second-generation integrase strand transfer inhibitor (INSTI) as part of preferred first-line regimens since 2017 [5,9,10]. Dolutegravir is characterized by high potency, favorable tolerability, and a high genetic barrier to resistance [9,10]. However, following its widespread scale-up in low- and middle-income countries, continuous surveillance of DTG resistance mutations and their impact on the efficacy of backbones combining DTG with nucleoside/nucleotide reverse transcriptase inhibitors (NRTIs/NtRTIs) is of paramount importance [11,12].

Sub-Saharan Africa remains the epicentre of the epidemic, accounting for more than two-thirds of all global cases [1,2]. As one of the most heavily affected country, the Democratic Republic of the Congo (DRC) is experiencing a generalized HIV epidemic [13], with molecular epidemiological studies identifying its capital, Kinshasa, as the historical epicenter of the global HIV-1 pandemic [14,15,16]. The viral landscape in the DRC is characterized by extensive genetic diversity, marked by the co-circulation of all known HIV-1 subtypes, high intra-subtype variation, and numerous complex recombinant forms [14,15,16,17]. An integrated biological and behavioral surveillance survey (IBBSS) conducted in 2012 among key populations across 11 cities in the DRC revealed that 21.5% of analyzed samples harbored antiretroviral (ARV) resistance mutations, with non-nucleoside reverse transcriptase inhibitor (NNRTI) resistance being the most prevalent [18].

Recently, Duhant et al. reported high rates of virological failure and HIV drug resistance mutations to NNRTIs (efavirenz, nevirapine) and NRTIs (abacavir, lamivudine) in Bukavu, South Kivu Province [19]. They also identified the L74I and G97A mutations in the integrase gene, which confer resistance to elvitegravir, a first-generation INSTI [19]. Although L74I and G97A are polymorphic and primarily affect first-generation INSTIs, their presence can occasionally lower DTG susceptibility when combined with major mutations or reflect baseline integrase variability prior to widespread DTG utilization [10].

Aligning with global guidelines, the DRC adopted DTG-based regimens as preferred first-line ART for people living with HIV in 2019 [13]. However, data on acquired drug resistance among patients failing DTG-based therapy in the country remain extremely limited. Such virological failure can occur in both treatment-naive individuals and treatment-experienced patients who have switched regimens.

Bunia, the capital of Ituri Province, shares borders with Uganda and South Sudan and experiences intense cross-border migration. Identified as a high-priority region for HIV control due to a prevalence exceeding the national average [20], Ituri delivers HIV care amidst post-conflict population instability, limited viral load monitoring infrastructure, and scarce adherence support interventions. However, data regarding the genetic diversity of circulating HIV strains and the prevalence of drug resistance mutations among patients on DTG-based regimens in this setting remain entirely lacking.

To address this knowledge gap, this study aimed to determine the prevalence of virological failure, characterize circulating HIV-1 subtypes, and identify drug resistance mutations among participants experiencing treatment failure on DTG-based antiretroviral therapy (ART) in Bunia, Democratic Republic of the Congo.

## 2. Materials and Methods

### 2.1. Study Setting and Study Population

This prospective cohort study was conducted among people living with HIV (PLHIV) receiving dolutegravir (DTG)-based antiretroviral therapy (ART) who experienced virological failure in Bunia, the capital of Ituri Province, Democratic Republic of the Congo (DRC), between January 2025 and January 2026. The study encompassed 20 healthcare facilities (HCFs) providing comprehensive HIV care in accordance with national guidelines based on the World Health Organization (WHO) public health approach for resource-limited settings [21]. Per WHO guidelines, virological failure was defined as a plasma viral load of 1000 copies/mL following at least six months of ART, confirmed by two consecutive viral load measurements taken three months apart with intervening adherence support [13].

### 2.2. Sampling Method

The subset of participants experiencing virological failure was derived from an initial cohort of 603 individuals. A non-random stratified quota sampling approach was employed to establish sample sizes across strata, followed by simple random sampling from the ART registers of each participating healthcare facility to select study participants. Initially, baseline plasma HIV viral load (VL) testing was conducted to determine the rate of viral suppression [8,22]. Participants with unsuppressed viral loads were then enrolled in a prospective cohort and followed for 3 to 6 months while receiving enhanced adherence counseling (EAC). Following this intervention period, follow-up VL testing was performed in accordance with WHO guidelines to identify cases of confirmed virological failure [13,21]. Confirmed virological failure was defined as a viral load exceeding 1000 copies/mL on two consecutive measurements taken 3 months apart, following adherence support provided after the initial unsuppressed result [13]. Baseline viral suppression outcomes from this primary cohort were recently published [23].

### 2.3. PCR Amplification and Sequencing

HIV-1 drug resistance genotyping was performed at the Central Public Health Laboratory (CPHL) in Kampala, Uganda. Viral RNA was extracted using the QIAamp Viral RNA Mini Kit (QIAGEN^®^, Courtabœuf, France) in strict accordance with the manufacturer’s instructions. Target amplification and library preparation followed the next-generation sequencing (NGS)-based HIV genotyping pipeline developed by the CPHL [24]. Briefly, primary RT-PCR was executed using the SuperScript™ IV One-Step RT-PCR System (Invitrogen^®^, Thermo Fisher Scientific, Vilnius, Lithuania) to amplify three target genomic regions across the pol gene. The integrase region (1021-bp amplicon) was amplified using the primer pair HIV_INT_1F (5′-AGAAGTAAACATAGTAACAGACTCACA-3′) and HIV_INT_1R (5′-ACAATCATCACCTGCCATCTGT-3′). The protease/reverse transcriptase region (2418-bp amplicon) was amplified using HIVPR_1F (5′-CAATTGTGGCAAAGAAGGGCAC-3′) and HIVRT_3R (5′-GACATTTATCACAGCTGGCTACTAT-3′).

A secondary nested PCR was then performed using the Platinum™ SuperFi II DNA Polymerase Kit (Invitrogen^®^, Thermo Fisher Scientific, Vilnius, Lithuania). This reaction yielded a 2116-bp amplicon spanning the PR/RT genes using primers HIVPR_3F (5′-CTGAAAGACAGGCTAATTTTTTAGGG-3′) and HIVRT_1R (5′-GTGCTGGTACCCATGACAGGTAG-3′), as well as a 1377-bp amplicon for the INT gene using primers HIV_INT_2F (5′-TATGGGGAAAGACTCCTAAATTTA-3′) and HIV_INT_2R (5′-AATCCTCATCCTGTCTAC-3′). Sequencing libraries were constructed from the purified amplicons using the Illumina DNA Prep Kit (Illumina, Inc., San Diego, CA, USA) according to the manufacturer’s instructions. Library quality control and fragment size distribution were evaluated on an Agilent 2100 Bioanalyzer using the High Sensitivity DNA Kit (Agilent Technologies, Santa Clara, CA, USA). Validated libraries were pooled and sequenced on the Illumina MiSeq platform using the MiSeq Reagent Kit v3 (300 cycles) to generate 150-bp paired-end reads.

Raw FASTQ files were assessed for sequencing quality using FastQC. Adapter and primer sequences were trimmed using Cutadapt, and the processed reads were converted into codon-frequency files using fastq2codfreq. Cleaned sequences were subsequently validated with the Los Alamos National Laboratory (LANL) HIV Sequence Database Quality Control tool (https://www.hiv.lanl.gov/content/sequence/QC/index.html; accessed on 11 July 2026) to screen for hypermutation, cross-contamination, and frame-shift mutations prior to down-stream assembly.

### 2.4. HIV-1 Subtyping and Phylogenetic Analyses

Initial HIV-1 subtyping was performed using the automated REGA HIV Subtyping Tool v3.47 (Genome Detective; https://www.genomedetective.com/app/typingtool/hiv; accessed on 12 July 2026). To confirm subtype assignments, query sequences were aligned alongside reference sequences using ClustalW implemented in Molecular Evolutionary Genetics Analysis X (MEGA X, version 10.2.6) [25]. The reference panel represented all established HIV-1 group M subtypes (A1, A2, B, C, D, F1, F2, G, H, J, K, and L), groups N, O, and P, circulating recombinant forms (CRFs), and a simian immunodeficiency virus outgroup (SIV_cpz), all retrieved from the Los Alamos National Laboratory (LANL) HIV Sequence Database (https://www.hiv.lanl.gov). Evolutionary distances were estimated using the Kimura 2-parameter (K2P) model, and phylogenetic trees were constructed in MEGA X using the Neighbor-Joining method. The reliability of the inferred phylogenetic topologies was evaluated with 1000 bootstrap replicates, with bootstrap values ≥ 70% considered supportive of node clustering.

### 2.5. HIV-1 Drug Resistance Analysis and GenBank Accession

The newly generated HIV-1 protease–reverse transcriptase (PRRT, accession numbers: QB047093-QB047145) and integrase (IN, accession numbers: QB047146-QB047196) sequences from Bunia were deposited in the NCBI GenBank database. HIV drug resistance (HIVDR) mutations were identified using the Stanford HIV Drug Resistance Database algorithm (HIVdb v10.2; https://hivdb.stanford.edu). Mutation frequencies were calculated for nucleoside reverse transcriptase inhibitors (NRTIs), non-nucleoside reverse transcriptase inhibitors (NNRTIs), protease inhibitors (PIs), and integrase strand transfer inhibitors (INSTIs). Phenotypic susceptibility was predicted based on HIVdb cumulative penalty scores and categorized into five resistance levels: susceptible (score < 10), potential low-level resistance (10–14), low-level resistance (score 15–29), intermediate resistance (score 30–59), and high-level resistance (score ≥ 60).

### 2.6. Calculation of the Genotypic Susceptibility Score (GSS)

Drug resistance mutations within the HIV-1 genome are a primary driver of virological failure, impairing both virological and immunological responses to ART. To evaluate the residual efficacy of prescribed regimens, a Genotypic Susceptibility Score (GSS) was calculated for each participant’s treatment combination based on the Stanford HIVdb genotypic resistance interpretation system [26]. The GSS reflects the total number of active or partially active antiretroviral agents in a regimen and serves as a key predictor of treatment outcomes [27].

Genotypic Susceptibility Scores (GSSs) were derived using the Stanford HIVdb resistance interpretation system. For each antiretroviral agent in a patient’s regimen, drug activity was assigned an individual value based on predicted resistance: 1.0 for susceptible virus, 0.5 for low-level or intermediate resistance, and 0 for high-level resistance. The total GSS for a given regimen was calculated as the sum of these individual drug scores (Table S1). Patients were stratified into three regimen-activity categories based on their cumulative GSS: <2.0, 2.0–2.5, and = 3. A total score of ≥3 reflects a fully active regimen, whereas scores of <3 indicate compromising drug resistance and reduced residual antiviral potency.

### 2.7. Statistical Analysis

Descriptive statistics were used to summarize participant characteristics and laboratory outcomes. Categorical variables were expressed as absolute frequencies and percentages. Continuous variables were assessed for normality and expressed as medians with interquartile ranges (IQRs) for non-normally distributed data, or means with standard deviations for normally distributed data.

## 3. Results

**Figure 1:**
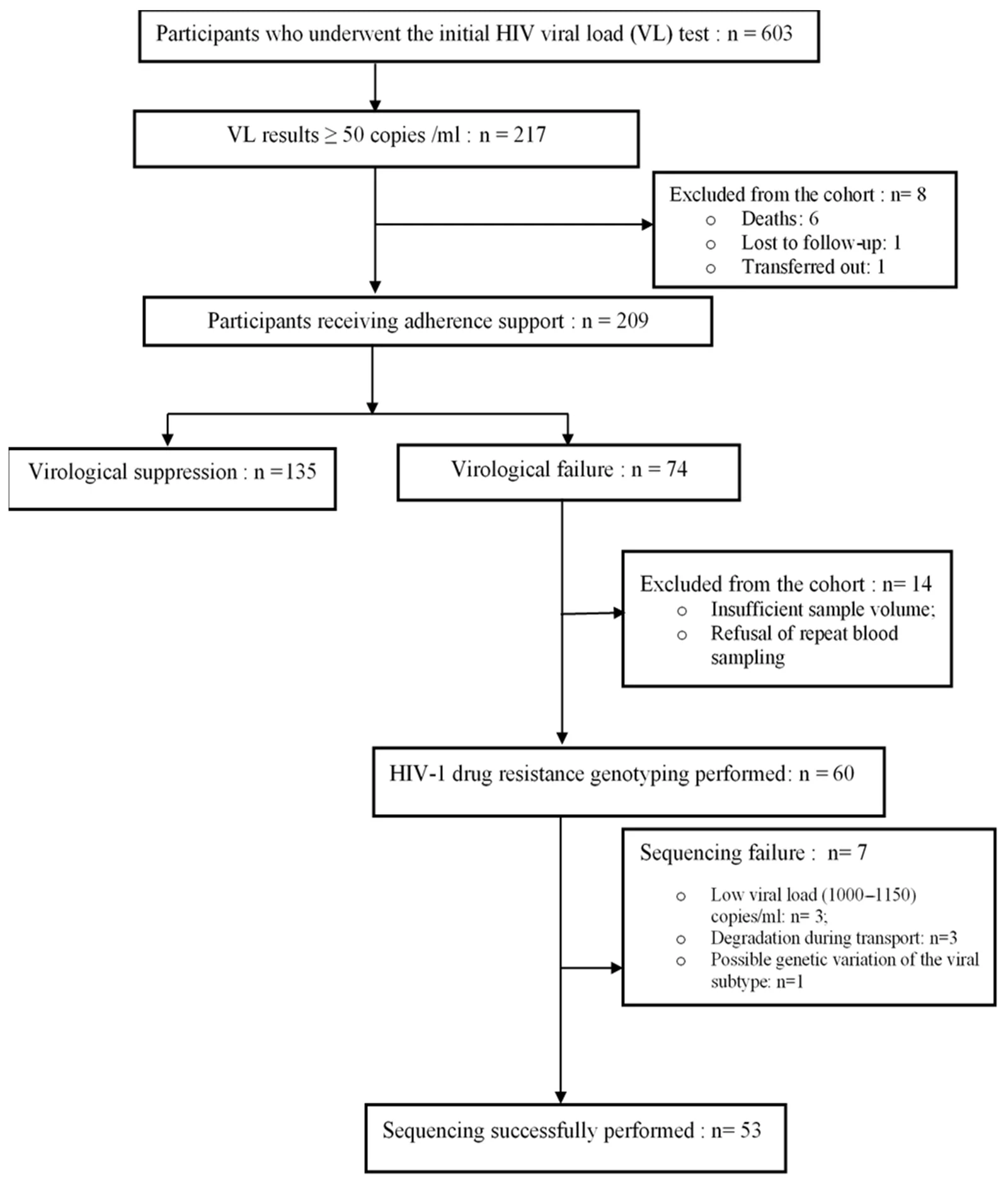
Flowchart detailing participant inclusion and the identification of virological failure in the study cohort.

Among the 603 people living with HIV (PLHIV) receiving antiretroviral therapy (ART), 74 experienced virological failure. The participant selection process, based on WHO criteria for virological failure, is summarized in Figure 1.

The study population was predominantly female (55.4%, *n* = 41), with a median age of 36 years (interquartile range [IQR]: 17.0; overall range: 5–78 years). Participants aged 28–37 years comprised the largest age group (37.8%, *n* = 28). Just over half of the participants were employed (51.4%, *n* = 38), while 48.6% (*n* = 36) were unemployed. Most participants (85.1%, *n* = 63) had completed formal education, with 47.3% (*n* = 35) reaching primary and 37.8% achieving secondary level (Table 1).

**Figure 2:**
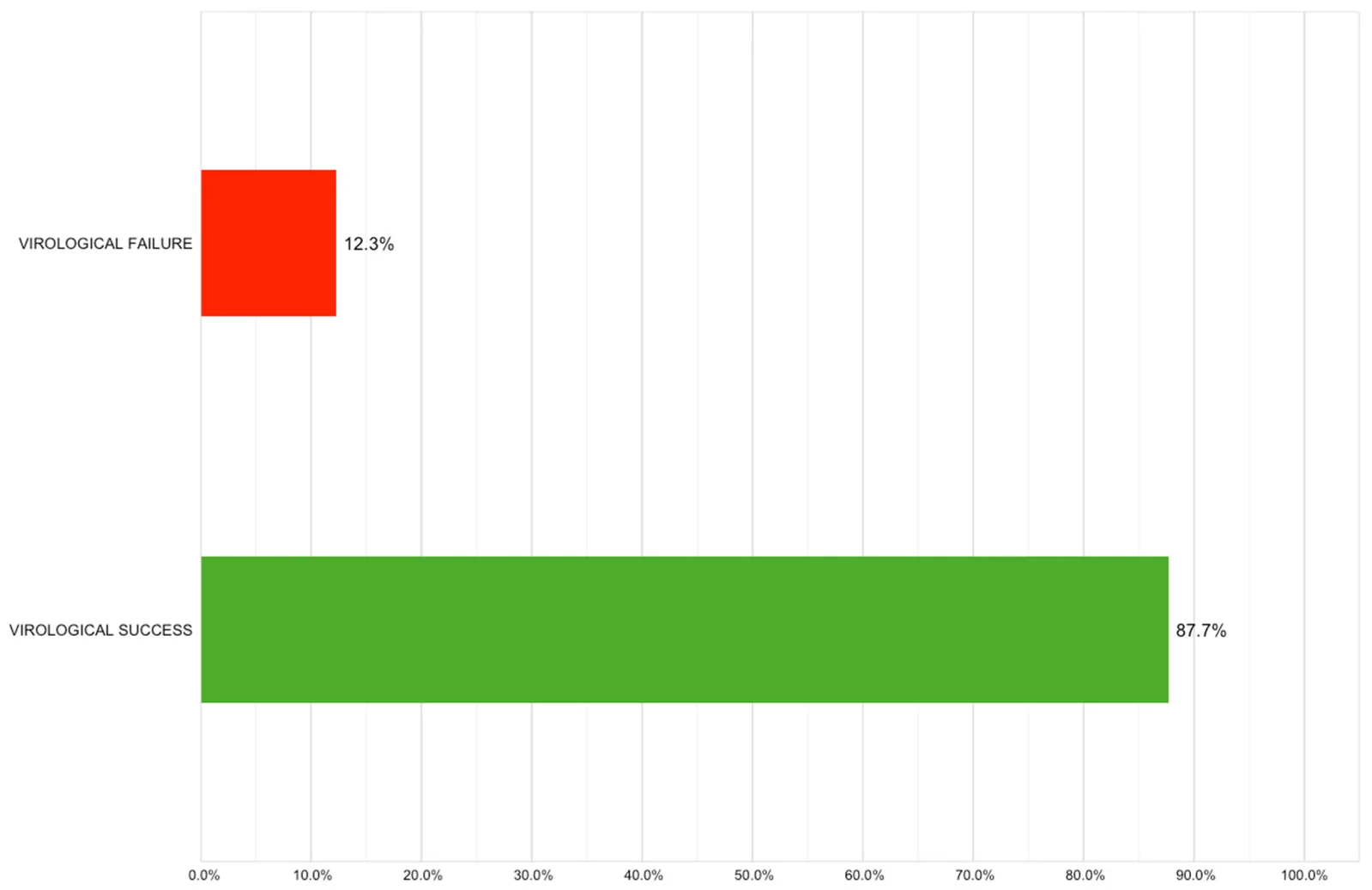
Prevalence of virological failure among individuals receiving first-line antiretroviral therapy in Bunia.

Furthermore, 29.7% (*n* = 22) of participants were single, and an equal proportion were cohabiting. The majority (54.1%, *n* = 40) were classified as WHO clinical stage III. The median duration on antiretroviral therapy (ART) across the entire cohort was 24 months. The overall prevalence of virological failure among individuals receiving ART was 12.3% (Figure 2).

**Figure 3:**
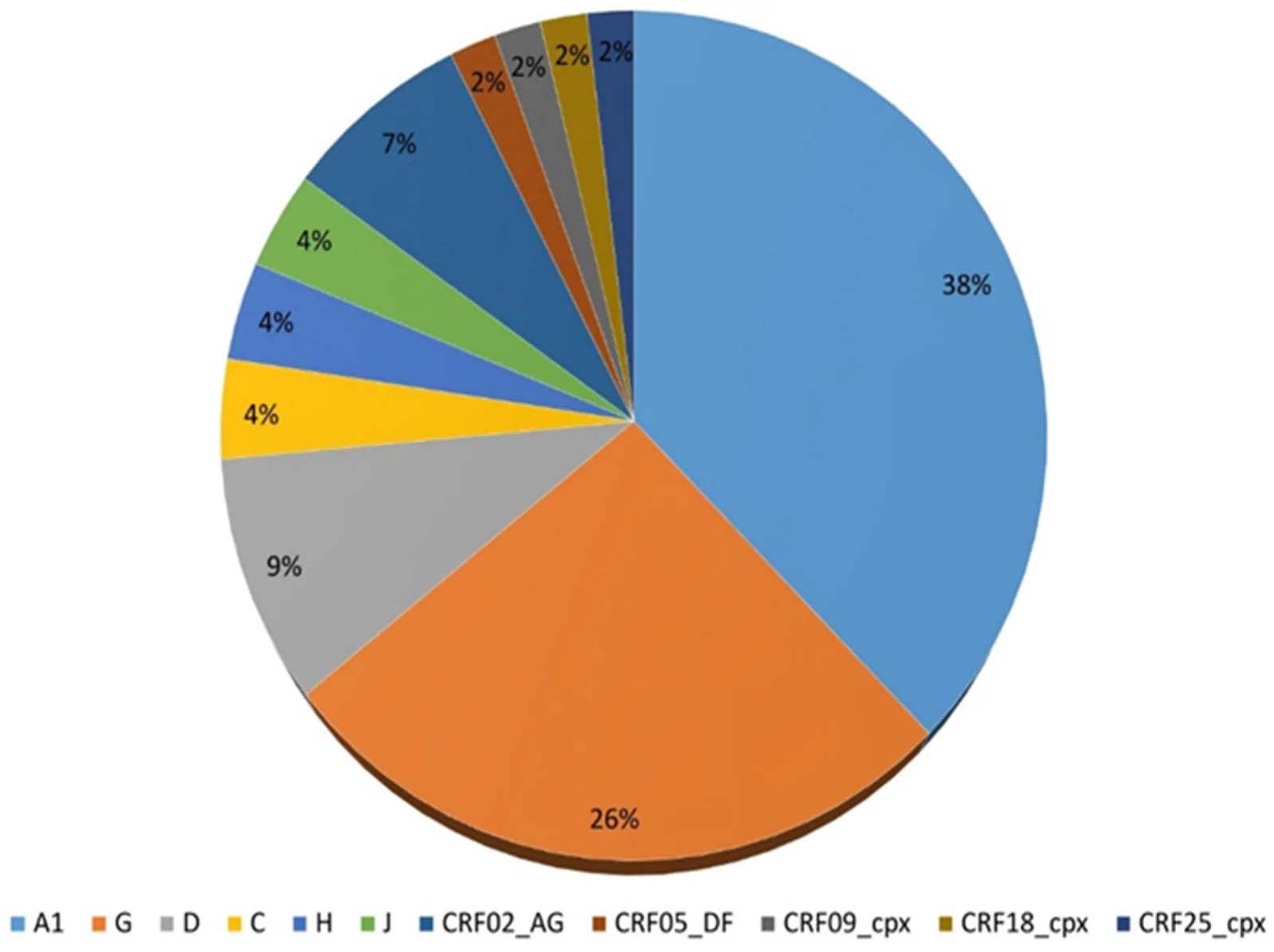
Prevalence and subtype diversity of HIV-1 among patients with virological failure on ART in Bunia.

A total of 53 sequences were successfully generated for both the protease–reverse transcriptase (PRRT) and integrase (IN) regions of the pol gene, yielding 51 paired PRRT–IN sequences. HIV-1 subtype analysis revealed a predominance of sub-subtype A1 (38%), followed by subtypes G (26%) and D (9%). Circulating and unique recombinant forms accounted for 16% of infections, with CRF02_AG being the most prevalent (Figure 3).

**Figure 4:**
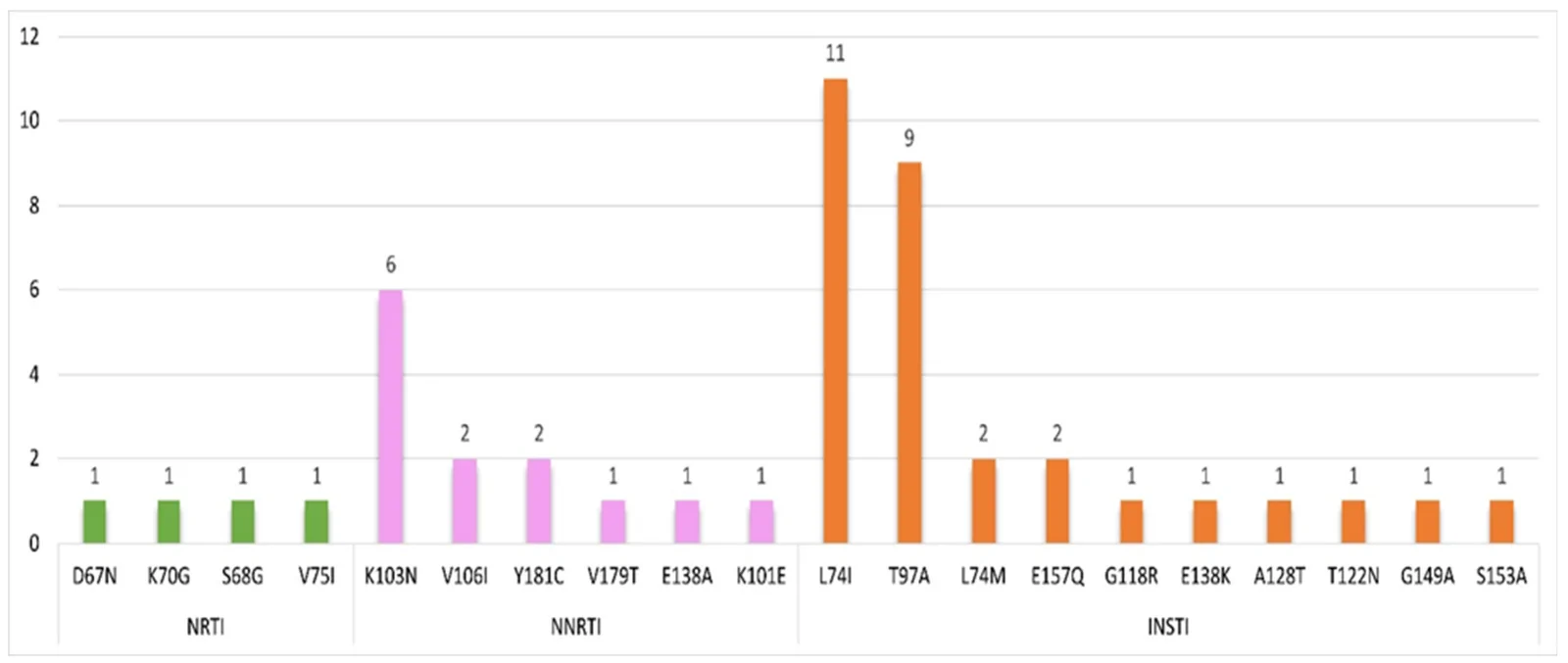
Prevalence of drug resistance mutations (DRMs) among participants experiencing virological failure in Bunia.

Analysis of drug-resistance mutations revealed that 26.4% (14/53) of the sequences harbored at least one mutation associated with resistance to reverse transcriptase inhibitors, while 33.9% (18/53) harbored at least one mutation associated with resistance to integrase strand-transfer inhibitors (INSTIs). Overall, we identified 10 major reverse transcriptase inhibitor resistance mutations, among which K103N was the most frequent (11.32%, 6/53) (Figure 4).

Likewise, 10 INSTI resistance-associated mutations were identified (Figure 4). Major INSTI resistance mutations were detected in a single patient, who harbored co-occurring G118R and E138K mutations. Minor resistance mutations were more widespread, dominated by L74I (20.8%, 11/53) and T97A (17.0%, 9/53).

No protease inhibitor (PI) resistance mutations were detected. Genotypic Susceptibility Score (GSS) analysis revealed that 98.0% (50/51) of patients had a fully active regimen (score of 3), whereas only 2.0% (1/51) had a compromised score (GSS = 1).

## 4. Discussion

To date, data regarding HIV-1 genetic diversity and drug resistance mutations among patients experiencing virological failure on DTG-based ART in northeastern Democratic Republic of the Congo (DRC) have been lacking. We report a 12.3% virological failure rate in Bunia, consistent with estimates from adult cohorts in Ethiopia (11.7% and 12.8%) [28,29]. This rate is higher than that observed in an Ethiopian pediatric cohort (10.6%) but substantially lower than the rate documented in Cameroon (25.5%) [30,31]. This failure rate is inextricably linked to the ongoing humanitarian crisis in Bunia, where active conflict severely limits routine virological monitoring. Conflict-driven population displacement, security-related supply chain disruptions, and healthcare infrastructure destruction continuously compromise and impair treatment adherence and continuity of care.

Antiretroviral drug resistance mutations (DRMs) are a primary driver of treatment failure, compromising ART efficacy while elevating the risk of opportunistic infections and transmitted drug resistance. The rapid accumulation of DRMs generates diverse viral quasispecies that expand HIV-1 genetic diversity. Characterizing this diversity is crucial for tracking epidemic evolution, refining prevention strategies, and optimizing ART regimens [32].

This study demonstrates extensive genetic diversity within HIV-1 group M, dominated by sub-subtype A1 (38%), subtype G (26%), and subtype D (9%). Recombinant forms, led by CRF02_AG, accounted for 16% of infections in Bunia. These results align with historical patterns of HIV-1 genetic diversity in DRC, where subtype A has predominated since the beginning of the epidemic [14,33,34,35]. However, local subtype distributions remain dynamic and continue to evolve [22]. Indeed, integrated biological and behavioral surveillance surveys among key populations revealed a shift from subtype A, G, and C dominance in 2012 to a subtype K-dominated profile in 2014 [14,34]. Regionally, our findings mirror the molecular profiles observed in East Africa (Kenya, Tanzania, and Rwanda) [35], but contrast with Central and West Africa (Cameroon, Angola, Central African Republic, Gabon, and Equatorial Guinea), where CRF02_AG plays a major role alongside subtypes A, G, D, H, J, C, and CRF01_AE [35,36]. They also differ distinctly from Southern Africa (South Africa, Zimbabwe, Mozambique, Malawi, Eswatini, and Botswana), where the epidemic is driven almost exclusively by subtype C [35].

Characterizing drug resistance prevalence, tracking the dissemination of resistant strains, and identifying associated risk factors among patients experiencing ART failure are essential for defining local resistance profiles and mitigating the burden of HIV-1 drug resistance [37]. Accordingly, we evaluated resistance mutations in individuals with virological failure to elucidate the drivers of treatment failure in this setting.

In this study, we report a low frequency of drug resistance mutations (DRMs) associated with nucleoside reverse transcriptase inhibitors (NRTIs) and integrase strand transfer inhibitors (INSTIs) among patients in Bunia experiencing virological failure on tenofovir-lamivudine-dolutegravir (TLD). Regarding NRTI-associated DRMs, only four mutations were identified across three patients. These mutations, D67N, K70G, S68G and V75I, do not independently impair susceptibility to the NRTI components (TDF + 3TC) of the TLD regimen. Among INSTI-associated DRMs, major mutations G118R and E138K co-occurred in a single patient. Together, these two mutations significantly reduce susceptibility to DTG in patients with virological failure [38]. Specifically, G118R is a nonpolymorphic mutation frequently reported in individuals experiencing virological failure and emergent resistance during DTG-based therapy, conferring an approximate 9-fold reduction in DTG susceptibility. E138K is common nonpolymorphic accessory mutation selected under RAL, EVG, CAB, and DTG exposure; while it does not reduce INSTI susceptibility on its own, it acts synergistically with other mutations, particularly at position 118 and 148, to markedly impair drug sensitivity [39].

The low DRM burden observed among individuals receiving DTG-based ART aligns with findings from Togo and Malawi, where nearly all patients experiencing virological failure harbored drug-sensitive viral strains despite high viral loads [40]. Conversely, our results contrast with data from the 2024 WHO HIV Drug Resistance Report, which highlighted that across several sub-Saharan African countries including Malawi, Mozambique, and Uganda, the prevalence of DTG resistance among individuals with unsuppressed viral loads ranged from 3.9% to 8.6% in routinely monitored populations and reached up to 19.6% among treatment-experienced individuals failing DTG-based regimens [41].

Crucially, integrase strand transfer inhibitor (INSTI) resistance mutations reported in the literature predominantly emerge in patients with pre-existing nucleoside reverse transcriptase inhibitor (NRTI) resistance [4]. Specifically, M184V and K65R confer high-level resistance to lamivudine (3TC) and tenofovir (TDF), respectively, resulting in functional dolutegravir (DTG) monotherapy over a compromised NRTI backbone. In our cohort, major NRTI resistance mutations were entirely absent; only K70G, which confers low-level resistance to TDF and 3TC, was detected in a single participant. This intact NRTI backbone explains the high Genotypic Susceptibility Scores (GSSs) observed, with 98.0% (50/51) of participants achieving a score of 3, reflecting robust regimen efficacy. Consequently, virological failure in Bunia appears to be driven primarily by suboptimal treatment adherence rather than drug resistance, a pattern consistent with our team’s previous findings [23]. Suboptimal adherence leads to subtherapeutic plasma DTG concentrations, which Schramm et al. demonstrated can trigger virological failure without selecting for resistance mutations [41]. The sole exception was a participant harboring co-occurring INSTI DRMs (G118R and E138K) and NRTI mutations (D67N and K70G), who exhibited a compromised GSS of 1.

Several accessory INSTI mutations were identified in our cohort, led by L74I (36.7%) and T97A (30.0%). In isolation, these secondary mutations do not impair DTG susceptibility or confer resistance to DTG-based regimens. However, they frequently represent naturally occurring genetic polymorphisms within non-B HIV-1 subtypes among ART-naïve individuals [42], or they can act synergistically with major mutations to diminish DTG sensitivity [43]. The presence of these accessory DRMs underscores the necessity of ongoing genomic resistance surveillance in settings like Bunia, where non-B HIV-1 variants predominate.

Certain limitations should be considered when interpreting our findings. First, the lack of detailed clinical histories, including prior antiretroviral exposure, precluded a comprehensive risk-factor analysis for DRM emergence, which would have further clarified resistance dynamics under current ART regimens. Second, missing codons resulting from partial sequencing failures across specific resistance-associated positions may have led to an underestimation of the true prevalence of resistance mutations.

## 5. Conclusions

Seven years after the widespread implementation of dolutegravir (DTG)-based antiretroviral therapy (ART) in Bunia, and despite persistent armed conflict restricting routine virological monitoring, our findings regarding therapeutic outcomes are highly reassuring. Although a notable rate of virological failure was observed, the prevalence of HIV-1 strains harboring drug resistance mutations (DRMs) to first-line antiretrovirals in the Democratic Republic of the Congo remains paradoxically low. These results confirm the high genetic barrier to resistance and the sustained clinical efficacy of DTG-based regimens in this setting. However, they also underscore the critical imperative to overcome logistical barriers to routine viral load monitoring and reinforce targeted adherence support. Addressing these structural challenges is essential to achieving the third UNAIDS 95–95–95 target and advancing toward the global elimination of HIV/AIDS as a public health threat by 2030.

## Figures and Tables

**Table 1:** Baseline characteristics of study participants.

Characteristics	*N* = 74	%
Gender		
Female	41	55.4
Male	33	44.6
Age group (years)		
<18	7	9.5
18–27	6	8.1
28–37	28	37.8
38–47	18	24.3
≥48	15	20.3
Employment Status		
Employed	38	51.4
Unemployed	36	48.6
Educational Level		
No formal education	6	8.1
Primary education	35	47.3
Secondary education	28	37.8
Higher education	5	6.8
Marital Status		
Single	22	29.7
Divorced/Separated	5	6.8
Married	18	24.3
Cohabiting	22	29.7
Widowed	7	9.5
WHO Clinical Stage		
Stage I	13	17.6
Stage II	19	25.7
Stage III	40	54.1
Stage IV	2	2.7
ART-Duration (Months)		
6–11	17	23.0
12–24	21	28.4
>24	36	48.6

## Data Availability

All data supporting the findings of this study are included within the article and its Supplementary Materials. HIV-1 nucleotide sequences generated in this study have been deposited in the NCBI GenBank database (accession numbers pending). Additional dataset files are available from the corresponding author upon reasonable request.

## Supplementary Materials

The following supporting information can be downloaded at: https://www.mdpi.com/article/10.3390/tropicalmed11100286/s1. Table S1. Calculation of the GSS for patients in Bunia.

## Abbreviations

The following abbreviations are used in this manuscript:

3TC	Lamivudine
AIDS	Acquired immunodeficiency syndrome
VF	Virological failure
ART	Antiretroviral therapy
ARV	Antiretroviral
CI	Confidence interval
CPHL	Central Public Health Laboratory
CRF	Circulating recombinant form
DRC	Democratic Republic of Congo
DRM	Drug resistance mutations
DTG	Dolutegravir
HCFs	Healthcare facilities
HIV	Human immunodeficiency virus
HIVdb	HIV database
IN	Integrase
INSTI	Integrase strand transfer inhibitor
IQR	Interquartile range
MEGA	Molecular Evolutionary Genetics Analysis
NNRTIs	Non-nucleoside reverse transcriptase inhibitors
NtRTIs	Nucleoside reverse transcriptase inhibitors
Pis	Protease inhibitors
PLHIV	People living with HIV
PRRT	Protease-reverse transcriptase
SDGs	Sustainable development goals
TDF	Tenofovir
TLD	Tenofovir-lamivudine-dolutegravir
UNAIDS	Join United Nations Programme on HIV/AIDS
VL	Viral load
WHO	World Health Organization
