# Supplementary material for: Low Prevalence of HIV-1 Antiretroviral Resistance Among Patients Experiencing Virological Failure on Dolutegravir-Based Regimens: A Prospective Cohort Study in Bunia, Democratic Republic of the Congo
Source: Trop Med Infect Dis. 2026 Oct 10;11(10):286. doi: 10.3390/tropicalmed11100286 (PMC13709768; doi:10.3390/tropicalmed11100286)
Supplement: Supplementary file 1 [file tropicalmed-11-00286-s001.zip › tropicalmed-4527870-supplementary.pdf]

Table S1. Calculation of the GSS for patients in Bunia

The GSS was calculated based on the resistance level assigned by the Stanford algorithm.

Thus, for a molecule with a level of:

- Susceptible: GSS = 1

- Low or intermediate resistance: GSS = 0.5

- High resistance: GSS = 0

Finally, the total GSS is the sum of the scores for all the molecules in the therapeutic combination

N°	Patients	Antiretroviral GSS component			Total GSS
		TDF	3TC	DTG	
1	Bunia-001	1	1	1	3
2	Bunia-002	1	1	1	3
3	Bunia-003	1	1	1	3
4	Bunia-004	1	1	1	3
5	Bunia-005	1	1	1	3
6	Bunia-007	1	1	1	3
7	Bunia-008	1	1	1	3
8	Bunia-009				3
9	Bunia-010	1	1	1	3
10	Bunia-012	1	1	1	3
11	Bunia-013	1	1	1	3
12	Bunia-014	1	1	1	3
13	Bunia-015	1	1	1	3
14	Bunia-016	1	1	1	3
15	Bunia-017*				
16	Bunia-018	1	1	1	3
17	Bunia-019*				
18	Bunia-020	1	1	1	3
19	Bunia-021	1	1	1	3
20	Bunia-022	1	1	1	3
21	Bunia-023	1	1	1	3
22	Bunia-024	0.5	0.5	0	1
23	Bunia-025	1	1	1	3
24	Bunia-026	1	1	1	3
25	Bunia-027	1	1	1	3
26	Bunia-028	1	1	1	3
27	Bunia-030	1	1	1	3
28	Bunia-031	1	1	1	3
29	Bunia-032	1	1	1	3
30	Bunia-033	1	1	1	3
31	Bunia-034	1	1	1	3
32	Bunia-035	1	1	1	3

33	Bunia-036	1	1	1	3
34	Bunia-037	1	1	1	3
35	Bunia-038	1	1	1	3
36	Bunia-039	1	1	1	3
37	Bunia-041	1	1	1	3
38	Bunia-042	1	1	1	3
39	Bunia-043	1	1	1	3
40	Bunia-044	1	1	1	3
41	Bunia-047	1	1	1	3
42	Bunia-048	1	1	1	3
43	Bunia-049	1	1	1	3
44	Bunia-050*				
45	Bunia-051	1	1	1	3
46	Bunia-052	1	1	1	3
47	Bunia-053	1	1	1	3
48	Bunia-054	1	1	1	3
49	Bunia-055	1	1	1	3
50	Bunia-056	1	1	1	3
51	Bunia-057*				
52	Bunia-058	1	1	1	3
53	Bunia-059	1	1	1	3
54	Bunia-060	1	1	1	3

N°	Patients	Antiretroviral GSS component			Total GSS
		ABC	3TC	DTG	
55	Bunia-029	1	1	1	3

*During sequencing, either a PRRT sequence or an integrase sequence was obtained, but not both fragments. Consequently, the GSS could not be calculated for these patients.
